# Tuning Electronic Structure and Optical Properties of Monolayered h-BN by Doping C, Cu and Al

**DOI:** 10.3390/molecules30010192

**Published:** 2025-01-06

**Authors:** Qun Li, Tengchao Gao, Kuo Zhang, Xiangming Che, Guolong Ni

**Affiliations:** 1 College of Metallurgy and Energy, North China University of Science and Technology, Tangshan 063210, China; liq@ncst.edu.cn (Q.L.); 13785052806@163.com (T.G.); z2540512276@outlook.com (K.Z.); 15256510486@163.com (X.C.); 2 Tangshan Key Laboratory of Special Metallurgy and Material Manufacture, Tangshan 063210, China

**Keywords:** h-BN, co-doping, electronic structure, optical properties, first-principles calculations

## Abstract

As a graphene-like material, h-BN has stimulated great research interest recently due to its potential application for next-generation electronic devices. Herein, a systematic theoretical investigation of electronic structures and optical properties of C-doped and Cu-Al co-doped h-BN is carried out by the first-principles calculations. Firstly, two different C-doped h-BN structures for the para-position and ortho-position are constructed. The results show that the C ortho-doped h-BN (BCN) structure with a band gap of 3.05 eV is relatively stable, which is selected as a substate to achieve the Cu-Al co-doped h-BN. Based on this, the effect of the concentration of C atom doping on the electronic and optical properties of Cu-Al co-doped BC_x_N (x = 0, 11.1% and 22.2%) is investigated. The results demonstrate that the band gap of Cu-Al co-doped BC_x_N decreases and the optical properties improve with the increase in C atom concentration. The band gap and static dielectric constant of Cu-Al co-doped BC_0_N, BC_1_N and BC_2_N are 0.98 eV, 0.87 eV and 0.23 eV and 2.34, 3.03 and 3.77, respectively. As for all Cu-Al co-doped BC_x_N systems, the adsorption peak is red-shifted, and the peak intensity obviously decreases compared to the undoped h-BN. Additionally, the Cu-Al co-doped BC_2_N exhibits the best response to visible light. This work will provide valuable guidance for designing and developing h-BN-based doping systems with good performance in the field of optical and photocatalysis.

## 1. Introduction

Since graphene was successfully isolated from graphite in 2004, two-dimensional (2D)-layered materials have stimulated great research interest [1]. As it is graphene-like and one of the most attractive 2D materials, h-BN has also attracted considerable attention due to its unique and remarkable properties, including excellent optical (high transparency, strong cathodoluminescence emission, etc.) and mechanical (highest stiffness and flexibility) properties, high thermal conductivity and chemical inertness [2,3,4,5,6]. These properties render it widely applicable in deep UV emitters and detectors, photoelectronic devices and photocatalytic and electrochemical fields, etc. [7,8,9,10,11].

The h-BN monolayer is isostructural to graphene, known as “white graphene”, and it has almost the same lattice structure (less than 2% lattice mismatch); however, the band gap of h-BN is very different from graphene. The graphene possesses a band gap of zero, while the h-BN nanosheet has a wide band gap of 5.9 eV, which greatly limits its application in the field of optoelectronics [12]. Thus, the reduction in band gap is urgently explored. Numerous studies reveal that heterogeneous atom doping is an effective strategy to achieve the regulation of electronic band structures and further change the optical and electrical properties. For example, Li et al. [13] found that the band gap significantly reduces from 8.44 eV of pure h-BN to 2.01 eV of model_5C (BCN-5) by introducing C atom into h-BN, ultimately resulting in a better adsorption property in separation engineering. Additionally, except for the non-metallic atoms doping (e.g., C [14], F [15] and Si [16]), the metal atom doping can also cause a similar phenomenon [17,18]. Legesse et al. [19] found that the band gap change for Al-doped h-BN is related to Al concentration. When Al concentration is 12.5%, the electronic band gap of the doped h-BN decreases to 4.10 eV from 5.95 eV in the original undoped bulk h-BN material. The significant bandgap reduction implies that Al-doped h-BN has a broad application prospect in deep UV optoelectronic devices. Liu et al. [20] reported that the doped composite (Cu-BN) is stable and exhibits high catalytic performance for CO oxidation. However, for the doped h-BN, although extensive studies have been conducted, they are usually about single nonmetal atom or metal atom doping, not the combination of nonmetal atom and metal atom doping. To sum up, it can be inferred from the aforementioned doped h-BN that studies on the co-doping of h-BN are not comprehensive [21,22,23]. Thus, tuning the band gap of h-BN by the co-doping of nonmental and metal atoms will be a potential way to improve its electronic structure and optical properties, which can combine the advantages of different doping atom and has profound meaning.

Herein, non-metallic C atoms, transition metal Cu atoms and inexpensive metal Al atoms were chosen as dopants to investigate the effect on electronic structure and optical properties of h-BN. A systematic study based on density-functional theory (DFT) method was carried out to analyzed the structural stability, electronic structure and optical properties of co-doping h-BN, including bandgap, the density of states (DOS), electron density and optical properties. This work will give a promising theoretical guidance for designing and optimizing h-BN doping systems.

## 2. Results and Discussion

### 2.1. The Stability of C-Doped h-BN at Different Positions

**Figure 1:**
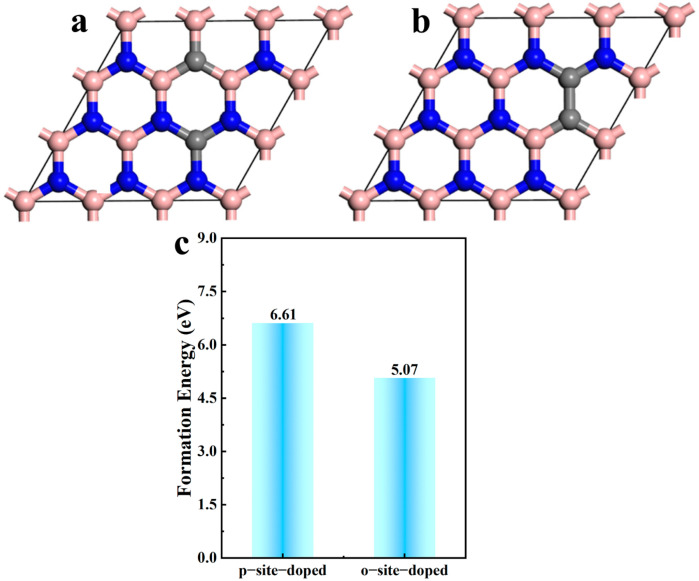
Structure of (**a**) C para-doped and (**b**) ortho-doped h-BN and (**c**) the corresponding formation energy.

C atom as dopants is firstly chosen because the diameter of C atom is the closest to the diameter B and N atom. Additionally, the C element is widely used to tune the electronic structure of h-BN. Thus, we chose the C atom for doping. Except for the C atom, we want to study the effect of transition metal elements and non-transition metal elements on electronic structure and optical properties of h-BN. Thus, the Cu atom, as the representative transition metal, and the cheaper non-transition metal Al atom were chosen. In order to explore the optimal doping position of C atoms in h-BN, two doping structures were constructed as shown in Figure 1. Figure 1a,b show that two carbon atoms are doped, respectively, in the para-position and ortho-position of a ring structure in h-BN. In order to evaluate the stability of the doped h-BN, the formation energy is calculated using the following equation [24,25,26]:

(1) $$E_{f}=E_{system}-E_{boron\;nitride}+m\mu_{X}-n\mu_{dopant}$$

where *E_system_* is the total energy of h-BN with substitutional doping, *E_boron nitride_* is the total energy of h-BN, *μ_X_* is the energy of a single free B/N atom and *μ_dopant_* is the energy of a single free dopant atom.

The obtained result of the formation energy is shown in Figure 1c. The formation energy for C-doped h-BN at the para-position and ortho-position is 6.61 eV and 5.07 eV, respectively, which demonstrates that the C-doped h-BN with the ortho-position is more stable.

### 2.2. Electronic Structure of C-Doped h-BN at Different Positions

**Figure 2:**
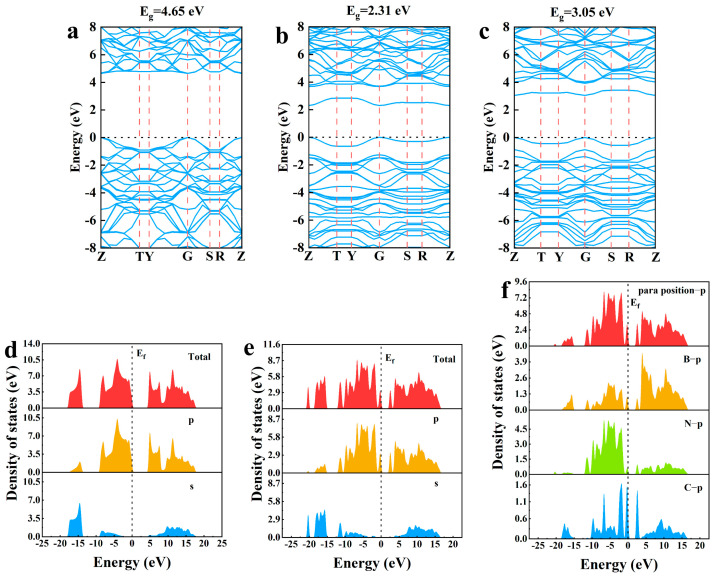
Band structure of (**a**) undoped h-BN and (**b**) C para-doped and (**c**) C ortho-doped h-BN. PDOS for (**d**) undoped h-BN and (**e**) C para-doped h-BN. (**f**) PDOS of p-orbital for B, N and C atoms.

The electronic structure of the crystal model, such as the band structure and the density of states (DOS), is usually calculated to explore the characteristics of electron motion and the energy states of electrons. The calculated results of band structure of undoped h-BN are shown in Figure 2a. The band gap for undoped h-BN is 4.65 eV. The calculated results of the band structure of two C-doped BN models are shown in Figure 2b,c. It can be observed that the band structures of two models do not pass through the Fermi level, indicating the forbidden band exists. The band gap for the para-position and ortho-position is determined to be 2.31 eV and 3.05 eV, respectively, which is obviously low compared with the undoped h-BN, indicating that the band gap of h-BN can significantly reduce when the C atoms with the same concentration are doped at different positions of h-BN. Furthermore, the result shows that the forbidden band width of the para-position is lower than that of the ortho-position, which means that the band gap of h-BN can be regulated more effectively by para-position C doping.

In order to further study the band gap reduction mechanism of C para-doped h-BN, the total density of states (DOS) and partial density of states (PDOS) for undoped h-BN and C para-doped h-BN are investigated and shown in Figure 2d,e. By comparison, it can be seen that a new peak appears near Fermi level in the DOS of C para-doped h-BN, which mainly comes from the contribution of p-orbital atoms. To further explore which atom contributes the most to p-orbital in the C para-doped h-BN, the contributions of each atom from the p-orbital are calculated in Figure 2f. From Figure 2a,b, it can be found that the bandgap reduction in C para-doped h-BN is mainly caused by the downshift of conduction band minimum (CBM), which can be contributed by the 2p orbital by comparing Figure 2d,e. In addition, Figure 2f shows that the state in CBM consists of B-2p, N-2p and C-2p orbitals, which declines the CBM and reduces the bandgap. Furthermore, the peaks of the conduction band appearing around 2.55 eV are mainly attributed to the contribution of C-2p orbitals and with minor contributions from the B-2p and N-2p orbital electrons.

### 2.3. The Stability of Cu-Al Co-Doped BC_x_N

**Figure 3:**
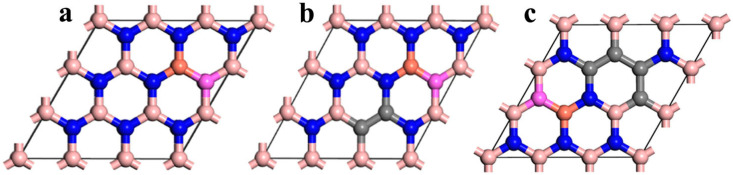
Structure of (**a**) Cu-Al co-doped BC_0_N, (**b**) Cu-Al co-doped BC_1_N and (**c**) Cu-Al co-doped BC_2_N.

Based on the above analyses, the relative stable structure of C ortho-doped h-BN (BCN) was used as substrates to explore the effect of Cu and Al co-doped BC_x_N on electronic structure and optical properties, where x = 0, 1 and 2 corresponds to the concentration of C atoms with 0%, 11.1% and 22.2%, respectively. As for the Cu-Al co-doped BC_x_N with different C atom concentrations, the Cu and Al atoms are doped in the ortho-position and replace the B atom and N atom, respectively. The corresponding structure is shown in Figure 3.

According to the formation energy equation in Section 3, the formation energy of co-doping with different C concentrations can be calculated, and the result is shown in Table 1. The formation energies of Cu-Al co-doped BC_x_N are 14.56 eV, 19.79 eV and 23.33 eV, respectively, when x = 0, 1 and 2. It can be seen that the formation energy of Cu-Al co-doped BC_x_N increases with the increase in C atom concentration.

### 2.4. Electronic Structure of Cu-Al Co-Doped BC_x_N

**Figure 4:**
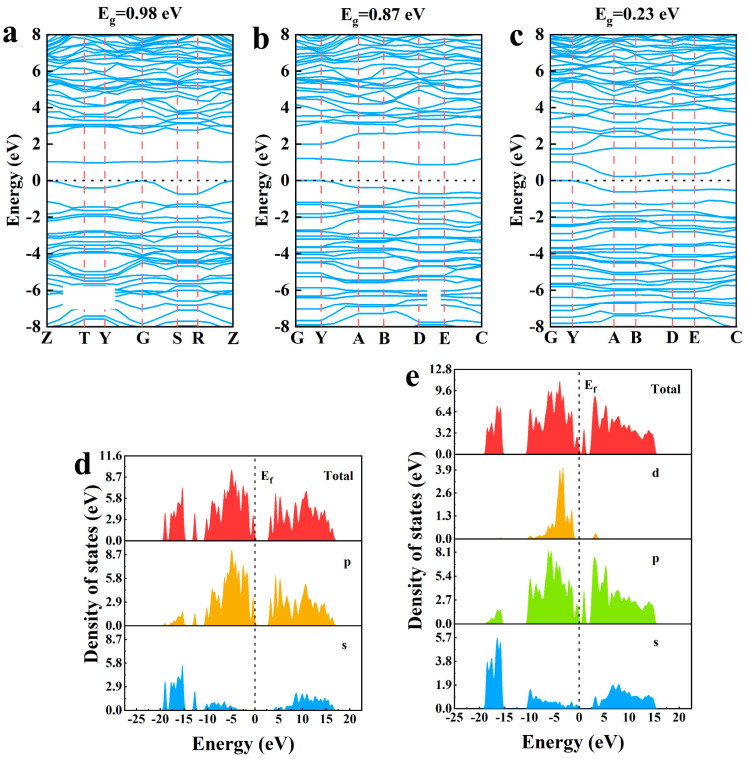
Band structure of (**a**) Cu-Al co-doped BC_0_N, (**b**) Cu-Al co-doped BC_1_N and (**c**) Cu-Al co-doped BC_2_N. PDOS for (**d**) C ortho-doped h-BN and (**e**) Cu-Al co-doped BC_0_N.

Figure 4a–c show the band structure and DOS of Cu-Al co-doped BC_x_N. When x = 0, 1 and 2, the band gaps are 0.98 eV, 0.87 eV and 0.23 eV, respectively. Compared with the C-doped h-BN mentioned in 3.2 part, the Cu-Al co-doped h-BN has a greater effect on the band gap of h-BN than C atoms. Additionally, the band gap of the system decreases with the increase in the concentration of C atom, implying that with the increase in the concentration of C atoms, the reduction effect of the Cu-Al co-doped BC_x_N system on the band gap is more obvious.

In order to further explore the reasons for the influence of Cu and Al atom doping on the band gap of h-BN, the DOS and PDOS were calculated for the Cu-Al co-doped h-BN (namely, BC_0_N), as shown in Figure 4e. Compared with the C-doped h-BN in Figure 4d, it can be found that a new d-orbital occurs in Cu-Al co-doped h-BN (Figure 4e), which is assigned to the inherent orbital property of metal atoms. A new peak in DOS and p-orbital can be observed on the right side of the Fermi level (Figure 4e), which is closer to the Fermi level than that of the C ortho-doped h-BN in Figure 4d, suggesting that the contribution of Cu and Al to p-orbitals is larger than that of the C atoms. Therefore, it can be concluded that the introduction of the new d-orbital and new peak in p-orbitals are the main reasons for the reduction in h-BN band gap.

### 2.5. Optical Properties

#### 2.5.1. Complex Dielectric Function

**Figure 5:**
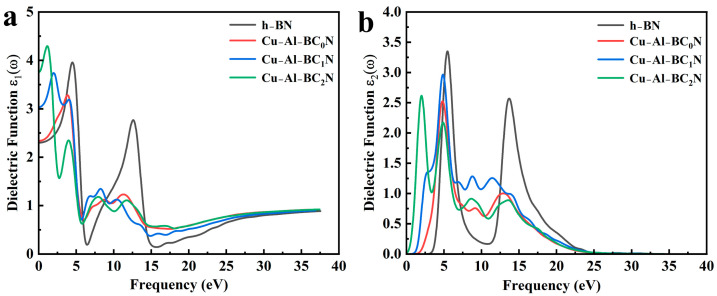
(**a**) Real parts and (**b**) imaginary parts of complex dielectric function for h-BN and Cu-Al co-doped BC_x_N.

The complex dielectric function ε(ω) describes the linear photovoltaic response to electromagnetic radiation, which is a key indicator of the material’s spectral characteristics [27,28,29]. The complex dielectric function ε(ω) consists of the real part ε_1_(ω) and the imaginary part ε_2_(ω). The formula of the dielectric function is ε = ε_1_(ω) + iε_2_(ω) [30]. The real part ε_1_(ω) is related to the dielectric energy loss or light absorption described by the material absorption coefficient. The real and imaginary parts of the complex dielectric function of Cu-Al co-doped BC_x_N are shown in Figure 5. As seen in Figure 5a, the intensities of high-frequency peaks for Cu-Al co-doped BC_x_N decrease with the increase in C atom concentration compared to pure h-BN, but their peak widths increase. The intensities of the low-frequency peaks of Cu-Al co-doped BC_x_N are red-shifted. The static dielectric constant of the undoped h-BN is 2.3. The static dielectric constants of Cu-Al co-doped BC_x_N are 2.34, 3.03 and 3.77, respectively. With the increase in C concentration, the static dielectric constant increases obviously, which indicates that the increase in C concentration enhances the ability of exciton decomposition into free charge and improves the utilization rate of light. The imaginary part ε2(ω) can provide the information about the light absorption and energy conversion efficiency [30,31]. It can be seen from Figure 5b that the intensities of high-frequency peaks for Cu-Al co-doped BC_x_N decrease with the increase in C atoms concentration compared to pure h-BN, and their peaks are red-shifted. The intensities of the low-frequency peaks of Cu-Al co-doped BC_x_N also decrease with the increase in C atoms concentration. The peak of the imaginary part of Cu-Al co-doped BC_x_N decreases slightly and shifts to the lower energy region because the band gap decreases after the addition of C, Cu and Al atoms.

#### 2.5.2. Light Absorption Spectrum and Reflection Spectrum

**Figure 6:**
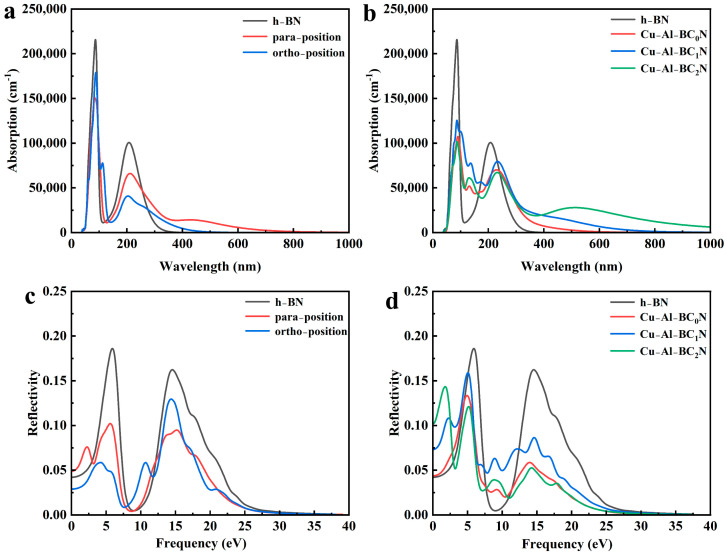
(**a**,**b**) Optical absorption of undoped h-BN and Cu-Al co-doped BC_x_N and (**c**,**d**) reflection of undoped h-BN and Cu-Al co-doped BC_x_N.

The absorption and reflection spectra of the C-doped h-BN at different positions and the Cu-Al co-doped BC_x_N are shown in Figure 6a–d. The higher the absorption rate means the more electrons transition from the ground state to the excited state, which further reflects the better optical responsiveness of the materials. As seen in Figure 6a,b, the absorption spectrum of the undoped h-BN can hardly be seen in the visible range of 360–780 nm, indicating that the undoped h-BN cannot absorb visible light. Noteworthily, with the increase in C atom concentration, the absorption spectrum red-shifts towards the visible light region due to the band gap reduction [32]. In addition, the Cu-Al co-doped BC_2_N exhibits the best response ability to the visible light region, and the absorption wavelength is increased more obviously. In addition, the higher reflectivity implies that the more the electrons transition to the excited state, then the more the electrons go back to the lower energy level by releasing energy. Figure 6c,d show the reflection spectrum. It can be found that the main reflection is in the range of 0–14.60 eV. When the photon energy is 0, the reflectivity of undoped h-BN and C para and ortho-doped h-BN is 0.042, 0.049 and 0.028, respectively. The reflectivity of Cu-Al co-doped BCxN (x = 0, 1 and 2) is 0.044, 0.073 and 0.103, respectively. Compared with the reflection spectra of the undoped h-BN and the C alone-doped h-BN, the reflection ability of Cu-Al co-doped BC_x_N increases significantly in the range of 0–1.5 eV, which reveals the Cu-Al co-doped BC_x_N system reflects infrared light more strongly.

#### 2.5.3. Complex Refractive Indexes and Extinction Coefficient

**Figure 7:**
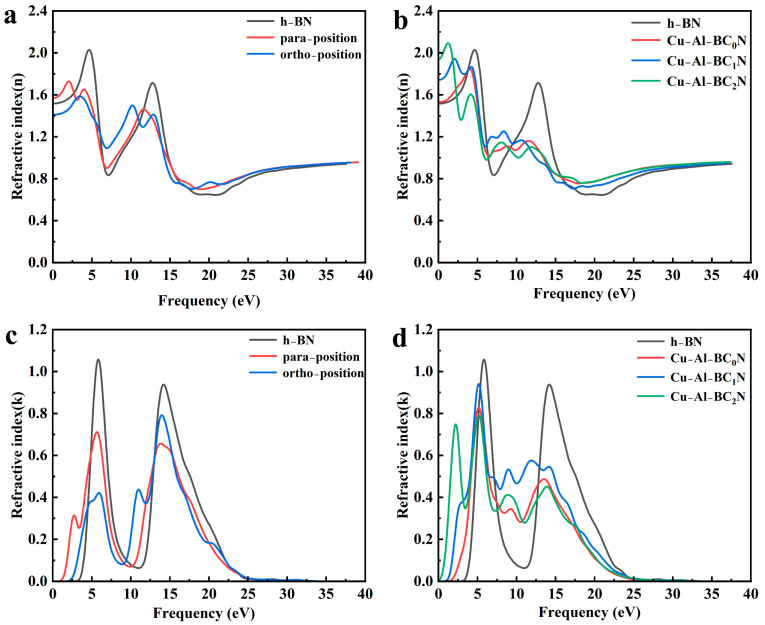
Complex refractive index of undoped h-BN and Cu-Al co-doped BC_x_N (**a**,**b**) n, (**c**,**d**) k.

Complex refractive indexes and extinction coefficients can be described with a complex refractive index as N = n + ik [30], where the real part (n) is the refractive index, and the imaginary part (k) is the extinction coefficient. It is well known that the curves of n and k for all h-BN systems are very similar to ε_1_(ω) and ε_2_(ω) of the complex dielectric function system [33]. As can be seen from the refractive index in Figure 7a,b, the refractive indexes of Cu-Al co-doped BC_x_N are better than that of C alone-doped h-BN in the high-frequency range (5–15 eV). However, the refractive indexes of Cu-Al co-doped BC_x_N are weaker than that of pure h-BN in the high-frequency range. However, their peak widths increase in the high-frequency range. It is observed that the refractive index peaks of C alone-doped h-BN and the Cu-Al co-doped BC_x_N in the low-frequency range (0–5 eV) are red-shifted compared to that of undoped h-BN. The red-shifted effect of Cu-Al co-doped BCxN are more obvious than that of C alone-doped h-BN in the low-frequency range. As shown in Figure 7a, the most refractive index of undoped h-BN, C para and ortho-doped h-BN is 4.55 eV, 2.02 eV and 3.44 eV, respectively. The most refractive indexes of Cu-Al co-doped BC_x_N are at about 3.89 eV, 1.96 eV and 1.28 eV. After the frequency of refractive index reaches the peak, with the increase in photon energy, the refractive index decreases rapidly and shows a trend of fluctuation and finally becomes stable. Figure 7c,d show the extinction coefficient. In Figure 7d, when the photon energy is 0–3 eV, the extinction coefficient increases with the increase in C atom doping concentration. In Figure 7c,d, it can be found that the intensities of the low-frequency peaks of C alone-doped h-BN and the Cu-Al co-doped BC_x_N decrease compared to that of undoped h-BN. In addition, the low-frequency peaks of Cu-Al co-doped BC_x_N shift slightly to low energy.

## 3. Computational Details

**Figure 8:**
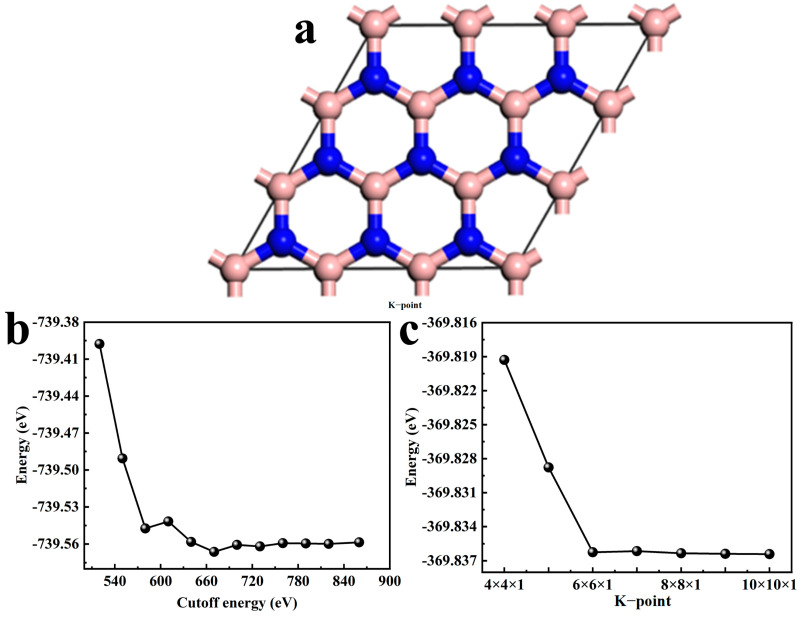
(**a**) Structure of h-BN supercell, (**b**) cutoff energy and (**c**) K-point convergence test of h-BN.

The first-principles calculations were carried out using the DFT-method-based CASTEP modules [34] to optimize the geometric structure and investigate the electronic structure and optical properties. The Perdew–Burke–Ernzerhof (PBE) functional within the formulation of the generalized gradient approximation (GGA) was selected to treat the exchange and correlation term. The crystal structure of h-BN (P6m2) with the lattice constants a = b = 2.50 Å and c = 6.77 Å was used to perform the geometric structure optimization. In order to eliminate the interaction between the bottom and top surfaces, the vacuum layer with a thickness of 10 Å was selected. A 3 × 3 × 1 supercell of the 2D h-BN monolayer composed of 18 atoms was created for calculation (Figure 8a). From Figure 8b, it can be found that when the cutoff energy (E_cut_) of h-BN is set to be 700 eV, the energy difference obtained by single-point energy calculations is small, indicating the energy is convergent. To ensure computational accuracy, E_cut_ = 720 eV was ultimately selected for calculations. Figure 8c shows the convergence test results for k-points. As observed, the total energy begins to converge when using a 6 × 6 × 1 k-point grid. Based on the convergence test results and computational accuracy, an 8 × 8 × 1 Monkhorst-Pack k-point grid was used to sample the Brillouin zone of h-BN monolayers.

## 4. Conclusions

In this paper, the structure stability, electronic structure and optical properties of the Cu-Al co-doped BC_x_N were studied by using the first-principles calculation method based on density functional theory. The band gap of undoped h-BN is 4.65 eV, and its absorption spectrum is mainly located in the ultraviolet region. By the doping of the C atom at different positions in h-BN, it can be determined that the C ortho-doped h-BN is more stable structure. Based on this, the effect of the concentration of C atom doping on the electronic and optical properties of Cu-Al co-doped BC_x_N (x = 0, 11.1% and 22.2%) is investigated. After Cu-Al co-doping, the band gap further decreases. Among them, the band gap reduction in Cu-Al co-doped BC_2_N is the most obvious, which is caused by the introduction of the new d-orbital and new peak in p-orbitals. Furthermore, as for all Cu-Al co-doped BC_x_N systems, the adsorption peak shows red-shifted and the peak intensity obviously decreases compared with the undoped h-BN. In addition, the Cu-Al co-doped BC_2_N exhibits the best response to visible light. The above results show the band gap and optical property of h-BN is greatly improved after doping, especially Cu-Al co-doped BC_2_N, which has profound meaning for designing the doped h-BN system and expanding its application in optoelectronic devices.

## Figures and Tables

**Table 1:** Formation energy of Cu-Al co-doped BC_x_N.

Materials	Cu-Al-BC_0_N	Cu-Al-BC_1_N	Cu-Al-BC_2_N
Formation energy/eV	14.56	19.79	23.33

## Data Availability

Data are contained within the article.
